# With a Little Help from My Friends: Group Orientation by Larvae of a Coral Reef Fish

**DOI:** 10.1371/journal.pone.0144060

**Published:** 2015-12-01

**Authors:** Jean-Olivier Irisson, Claire B. Paris, Jeffrey M. Leis, Michelle N. Yerman

**Affiliations:** 1 Rosenstiel School of Marine and Atmospheric Sciences, University of Miami, Miami, FL 33149-1098, United States of America; 2 Sorbonne Universités, UPMC Univ Paris 06, CNRS, Laboratoire d’Océanographie de Villefranche (LOV), 06230 Villefranche-sur-Mer, France; 3 Australian Museum Research Institute, Sydney, NSW 2010 Australia; 4 Institute for Marine and Antarctic Studies, University of Tasmania, Hobart, TAS 7004, Australia; University of California Santa Cruz, UNITED STATES

## Abstract

Theory and some empirical evidence suggest that groups of animals orient better than isolated individuals. We present the first test of this hypothesis for pelagic marine larvae, at the stage of settlement, when orientation is critical to find a habitat. We compare the *in situ* behaviour of individuals and groups of 10–12 *Chromis atripectoralis* (reef fish of the family Pomacentridae), off Lizard Island, Great Barrier Reef. Larvae are observed by divers or with a drifting image recording device. With both methods, groups orient cardinally while isolated individuals do not display significant orientation. Groups also swim on a 15% straighter course (i.e. are better at keeping a bearing) and 7% faster than individuals. A body of observations collected in this study suggest that enhanced group orientation emerges from simple group dynamics rather than from the presence of more skilful leaders.

## Introduction

Many animals move in groups and this is known to dilute predation, make mating easier, and help detect sources of food [1]. In addition, theory predicts animals should navigate towards a target better when in groups than as isolated individuals. The proposed mechanisms involved range from following the individual with the best navigational abilities to averaging out errors among members of the group [2–4]. Empirical evidence of such navigational benefits is convincing now [5–11] (but see [12] for a counter example). Yet, all tests were conducted on homing birds, almost all of them on pigeons, whereas the initial reasoning was developed for fish schools [13]. A corollary of the above-cited theories is that navigational accuracy should increase with the size of the group. This was also tested in birds: a phylogenetic study showed that species migrating over longer distances travel in larger groups [14].

In the ocean, most coastal organisms produce dispersive larvae which spend from a few hours to a few months in open water. This dispersal episode is a period of extremely strong selection (survival rates of fish larvae, for example, are in the order of 10^-5^ over a few weeks [15]). At the end of this phase, these pelagic, larval organisms must find a habitat, often very specific, near the coast (the settlement phase) and metamorphose into demersal juveniles that later integrate the adult population (the recruitment phase) [16]. Settling on a lesser quality habitat during (such as one with high competition [17]) or delaying metamorphosis until a good one is found [18] can reduce survival at the juvenile stage, increasing selective pressure further. Orientation during this pelagic phase is therefore critical, particularly at the end, making pelagic larvae good candidates for study of orientation behaviour.

At the end of the pelagic phase, the larvae of fishes, decapod crustaceans, and cephalopod mollusks, at least, are quite motile [19]. For example, late-stage tropical marine fish larvae can sustain speeds of 15 cm s^-1^ for several days, hence travelling tens of kilometres without rest or food (review in [16]). This movement is also oriented and larvae of fish, crabs, lobsters, and even corals respond to chemical cues and possibly coastal sounds while they settle [20–22]. In spite of their small size and incomplete development, these organisms seem to have the motor, sensory, and cognitive equipment for complex navigation behaviour.

In addition to being a potential test-case for navigation studies in the ocean, the pelagic phase of coastal species has long been recognised as an important determinant of adult stocks sizes [23] and, more recently, of the demographic and genetic structure of coastal meta-communities [24]. A century of research has uncovered various determinants of recruitment success such as the abundance and quality of food and the importance of ocean currents [25]. Increasing focus is now put on the behaviours of larvae that affect dispersal and connectivity between adult populations, in particular because many numerical models that include behaviour result in order of magnitude changes in predicted rates and spatial patterns of settlement [26–28].

This study is the first test of group orientation in a Class other than birds and focuses on marine larvae. If groups orient significantly better than isolated individuals, as theory predicts, this would have important ecological consequences for recruitment success and connectivity. This test was carried on settlement-stage coral-reef fish larvae, which are known to be very active [16]. We recorded their orientation using two techniques and compared the behaviour of isolated individuals and groups of about ten larvae.

## Materials and Methods

### Target location and species

**Fig 1:**
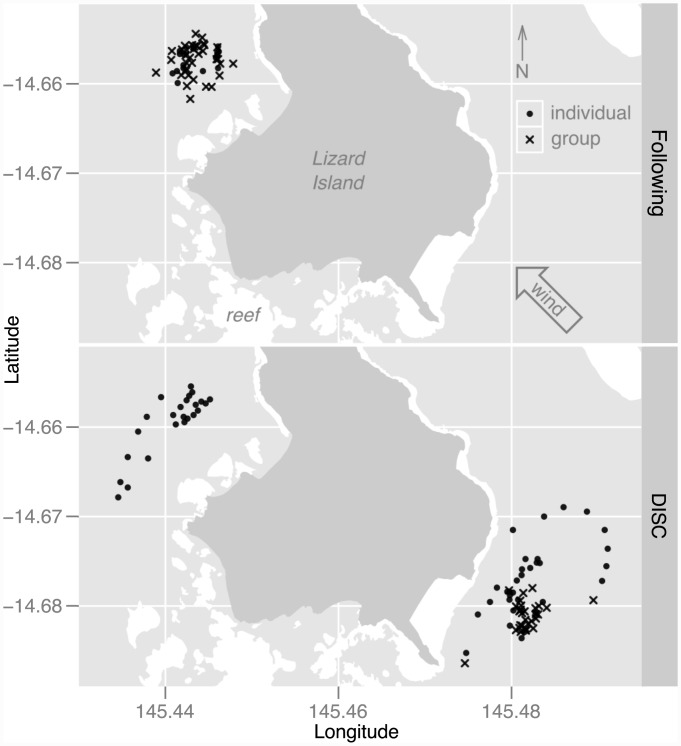
Observation locations around Lizard Island, Great Barrier Reef. Symbols mark the starting points of the observations by scuba divers (top) and in the Drifting In Situ Chamber (DISC; bottom). Bullets are observations of isolated individuals, crosses are groups. The direction of prevailing winds, defining the windward and leeward sides of the island, is shown in the top panel.

Observations were carried out in Nov. and Dec. 2008, around Lizard Island, Great Barrier Reef, Australia (Fig 1), where previous information on the orientation of individual fish larvae is available (summarised in [29]). This research was carried out under Research Permit G07/23641.1 from the Great Barrier Reef Marine Park Authority and Australian Museum Animal Care and Ethics Committee Approval 07–02.

Light traps were set outside the eastern (windward) and western (leeward) reefs, in the evening, to capture settling fish larvae. The catch was collected around 06:00 the next morning. Fishes were sorted out taxonomically, placed in 20 L, covered, white plastic buckets, and studied on the day of capture. Water in the buckets was changed every hour to avoid hypoxia or over-heating.

The target species was the common pomacentrid damselfish *Chromis atripectoralis*. It orients cardinally around Lizard Island [29], light traps catches are relatively high, and it lives in groups on small coral heads once settled, so it is likely to form shoals in open water (for which there is preliminary evidence [30]).

*Chromis atripectoralis* larvae caught by light traps are 7–10 mm in standard length and pigmented. Depending on the authors, such fishes would be called larvae, pre-settlement larvae, or post-larvae. We will not debate these terms here and simply designate the fishes under study as “larvae” as they are not yet settled on the reef.

Larvae were transported in a small motor boat to within 500 m to 1 km of the reef edge. Depending on wind direction and sea state, observations occurred on the eastern side of the island, over a 35 m-deep bottom covered by Halimeda meadows, or off the western side, over a 20 m-deep, bare sand, bottom (Fig 1). Observation times during the day ranged from 09:00 to 15:30, approximately. Two observation methods were used: following larvae by divers and photographing larvae in a drifting chamber.

### Scuba diver following

We followed the procedure introduced by [31], which has been used successfully with several species in various places (including *C*. *atripectoralis* in Lizard Island) and acts as the reference method.

Two scuba divers take a larva to a depth of 5 m in a container. The divers face each other and release the larva in a random direction. The first diver follows the larva from a distance of about 1 m. The second follows the first and records the larva’s bearing (to the nearest 5°) and depth (to the nearest 0.1 m) every 30 s, as well as the reading of a calibrated flowmeter every 5 mins (which provides average swimming speed). An observation run lasts for 10 mins or until the larva is lost. The motor boat circles at idle speed as far as possible around the divers’ bubbles to ensure their safety and avoid creating a directional sound cue for larvae. The time and position are recorded at the start and end of each observation run.

The same procedure was used to study the orientation of groups of larvae, except that 10–12 larvae were released simultaneously. The bearing recorded was that of the individual at the centre of the group. If the group split, the most numerous remaining group was followed.

### Drifting In Situ Chamber

The Drifting In Situ Chamber (DISC) allowed unmanned observation of orientation, at higher frequency than scuba diver following, albeit in a more restricted environment. The instrument is fully described in [32] and S1 Fig. The device comprises a cylindrical frame with a circular observation chamber at the top (38 cm diameter) and an electronic housing at the bottom. The frame is linked to a cruciform drogue, which keeps the device locked in the current and forces it to rotate. The housing contains an upward-looking camera which takes a picture of the chamber every 2 s, revealing the position of the larva in silhouette. The picture is geo-referenced cardinally using an electronic compass and a Global Positioning System (GPS). Because it is made of acrylic, the device is almost neutrally buoyant, transparent to sound, and inconspicuous underwater. The observation chamber is made of translucent mesh on the top and side wall and is therefore open to environmental signals (sound, chemicals, light, etc.).

The larva was placed in the chamber at the surface. Then the device slowly sank to its fixed depth of 9 m, the average depth of free-swimming *C*. *atripectoralis* observed in previous studies [33], and the larva was left to acclimate for 5 mins. During that time, the boat motored upwind and the motor was then switched off during the subsequent 10 mins of observation. Finally the boat rejoined the surface float, the DISC was hauled to the surface and the larva replaced.

The image-processing software described in [34] was slightly modified to accept high-resolution still images instead of video as input. A graphical user interface allows clicking on the larva (or the individual at the centre of the group of larvae) to record its position relative to the chamber. Using the time-synchronised digital compass record, the angle between the north, the centre of the chamber, and the position of the larva can be computed and gives the bearing of the larva. Bearings are rounded to the nearest 5° to match diver following data.

Because the DISC rotates, a larva keeping a bearing would counter the rotation whereas a larva artefactually attracted to a particular structure of the instrument would rotate with it. In 9 out of 149 deployments, the trajectory of the larva clearly showed it rotated with the instrument (positions very concentrated around one spot in the chamber but spread out across all bearings cardinally). Those deployments were discarded.

Images were subsampled at 10 s interval to provide independent position records. Settlement stage *C*. *atripectoralis* routinely swim at about 25 cm s^-1^ *in situ* [35] which would allow them to easily move around the 38 cm diameter chamber in 10 s.

### Statistical analysis

The data recorded are bearings: swimming directions when following larvae and bearings of positions in the DISC’s chamber. Bearings are recorded in magnetic degrees, which varies from degrees true by about 7° in the study area. Detecting cardinal orientation involved two analysis steps, typical in circular statistics [36].

First, *directionality* was assessed *within* each run, using the Rayleigh test (first-order analysis). Directionality represents the ability to swim in a straight line, i.e. to keep a bearing. This test provides a statistic, *r*, which is close to 0 when bearings are uniformly distributed around a circle or close to 1 when bearings are concentrated around a unique direction, and a *p*-value which assesses the significance of this concentration.

If larvae all swam directionally but towards different directions, it would suggest that they are capable of keeping a bearing but do not of orienting towards a common goal. *Orientation* was assessed by a test *across* runs: the previously significant within-run mean bearings were used as data in a second-order Rayleigh test.

Circular mean and standard deviation are reported. The circular standard deviation follows [37], converted to degrees, and is analogous to the standard deviation of linear data for unimodal distributions.

Comparisons of the strength of directionality (within-run *r*) between individuals and groups (or between observation methods, locations, etc.) were done through beta regression, because *r* is bounded in [0,1] [38]. Residuals never showed strong heteroscedasticity or non-normality (as per the Shapiro-Wilk test).

To test the effect of swimming in groups on orientation, the distributions of within-run mean bearings were compared between groups and individuals. The distributions were compared with the non-parametric Watson’s U^2^ test and the across-runs means were compared with the parametric Watson-Williams F-test, when applicable.

For diver following, within-run mean depths and speeds were also compared between groups and individuals. Parametric tests (t-test for means, F-test for variances) were used. Again, residuals never showed heteroscedasticity or non-normality.

Although *C*. *atripectoralis* were expected to orient cardinally [29], orientation with respect to the direction of the coast and the sun was inspected. Indeed, orientation towards the coast is the usual hypothesis for settlement-stage marine larvae and could be associated with many coastal cues (sounds, odours, etc.). Use of the sun as a compass is emerging as a potential mechanism for large scale orientation in the early life stages of fish [39, 40] and view of the sun is known to influence orientation of *C*. *atripectoralis* larvae around Lizard Island [35]. To inspect such directional cues, a Rayleigh test on the *difference* between the bearing of the larva and the bearing of the cue was used. As an example, in that setting, if larvae orient towards a cue the test is significant and the mean difference in bearings is close to 0°. Finally, the direction of the sun is easier to detect in the morning and evening because the sun is lower in the sky. Therefore, the relationship between the strength of directionality (within-run *r*) and the zenith (angle of the sun from the vertical) was also inspected, through beta regression.

All analyses were performed in R 3.1.2, with packages circular 0.4–7 for circular statistics, plyr 1.8.1 for data handling and ggplot2 1.0.0 for graphics.

## Results

### Summary of observations

A total of 140 runs were recorded. Larvae kept a bearing in 136 (97%) of them (significant directionality; numbers in columns ‘*n*’ and ‘*n* dir.’ of Table 1). Most following runs lasted the full 10 mins (average duration was 9.5 mins).

**Table 1:** Statistics on the orientation of larvae separated between experimental treatments (individual: ind or group), methods (Following: Fol or DISC), and locations (East: E, West: W, or pooled: –). Columns are: number of runs, total (*n*) and directional (*n* dir.); average within-run strength of directionality ($\bar{r}$ ∈ [0,1], higher means more directional); orientation mean bearing ± standard deviation; concentration of bearings across-runs (*r* ∈ [0,1]) and its *p*-value (*p*<0.05 denotes significant orientation).

		Within run		Across runs		
	*n*	*n* dir.	$\bar{r}$	bearing	*r*	*p*-value
ind Fol W	18	17	0.85	165 ± 91°	0.28	0.26
ind DISC E	32	31	0.63	122 ± 115°	0.13	0.59
ind DISC W	24	23	0.69	352 ± 147°	0.04	0.97
ind DISC –			0.65	111 ± 133°	0.07	0.79
group Fol W	35	35	0.93	182 ± 62°	0.56	< 10^-5^
group DISC E	31	30	0.79	193 ± 65°	0.52	< 10^-3^

For single individuals, the distribution of bearings is similar in all comparisons: between east and west in the DISC (Watson, U^2^ = 0.04, *p*>0.1), between DISC and following on the western side (Watson, U^2^ = 0.05, *p*>0.1), etc. The mean strength of directionality ($\bar{r}$) is also consistent across locations but often lower in the DISC than when followed by divers (beta regression with method and location as factors; no effect of location, z = −1.134, *p* = 0.257; significant effect of method, z = −4.341, *p*<10^-4^). An in-depth analysis of many more datasets [29] shows that, for individuals, (i) orientation is consistent across locations and methods and (ii) directionality should be inspected within methods. This guides similar comparisons for groups.

### Group swimming

**Fig 2:**
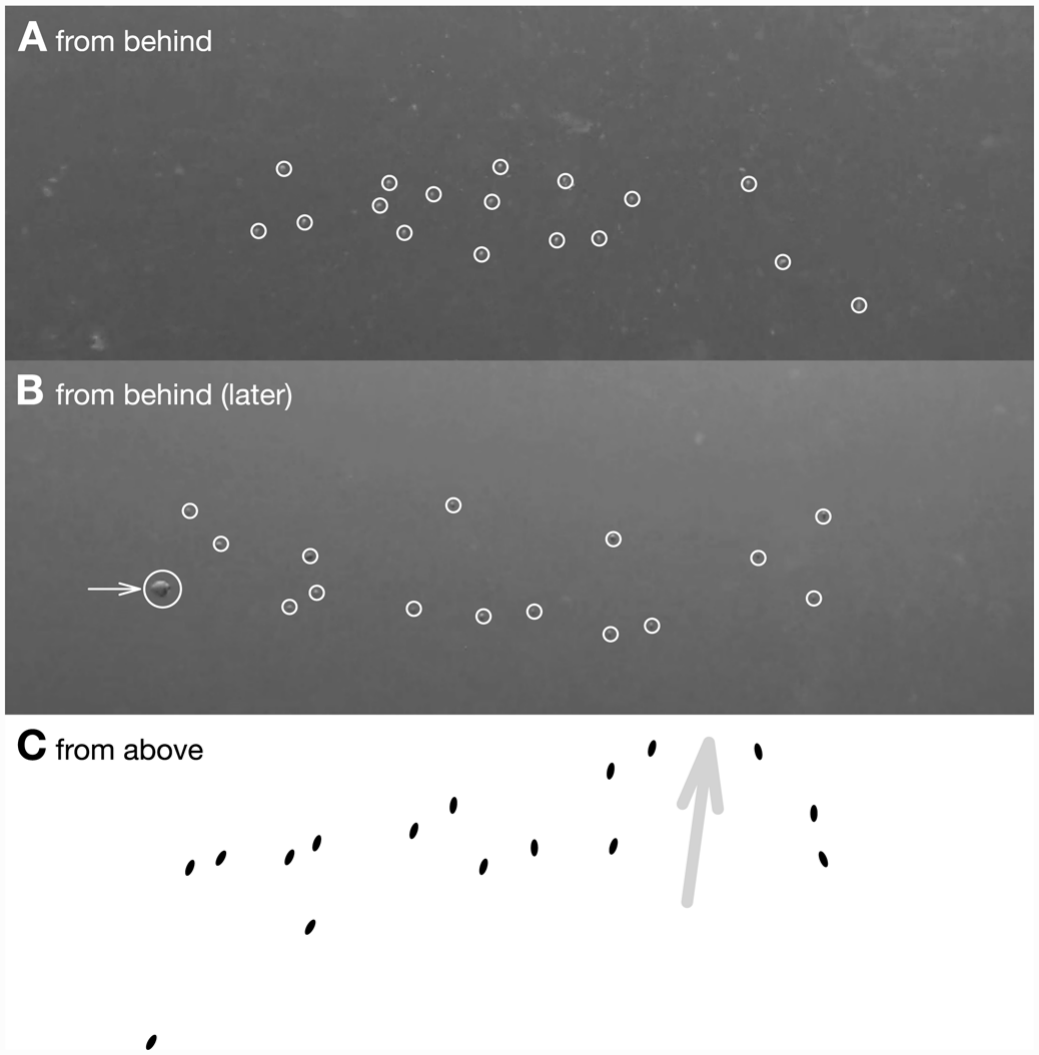
Configuration of groups of *C*. *atripectoralis* swimming. A and B: frames of a video shot while following a test group of 17 larvae (from behind); larvae are circled for clarity (video by C. Paris and R. Paris provided as supplementary material S1 Video). In B, the arrow points an individual on the verge of disconnecting from the group. C: sketch of what the group in B would typically look like from above. The grey arrow shows the direction of swimming recently initiated by a few individuals on the right side of the group.

Groups of *C*. *atripectoralis* stay cohesive both in the DISC and while followed in open water. In the DISC they move synchronously as a dense shoal. In open water, they spread along the horizontal, forming a slightly bent arc when seen from above (Fig 2). In both cases, changes of direction are initiated by one or a few individuals, almost immediately followed by the others. The leading individuals do not seem to be the same across the 10 mins of observation.

In open water, some individuals disconnect from the group under observation by divers. Most commonly, one individual would disconnect from the group from time to time (Fig 2B) but in 13 runs (out of 35) four to six individuals disconnected from the main group at once. The decrease in number of larvae observed is mostly steady: a median number of 12 larvae are released, 10 remain after 2 mins, 7 after 5 mins, and 5 after 10 mins. Splitting observations between runs in which less or more than 5 individuals remain at the end reveals no difference in the strength of directionality (beta regression, z = −0.05, *p* = 0.96), distribution of bearings (Watson, U^2^ = 0.0794, *p*>0.1), swimming depth (t-test, t = −1.2, *p* = 0.2), or swimming speed (t-test, t = 1.2, *p* = 0.2). So all data are used further.

**Fig 3:**
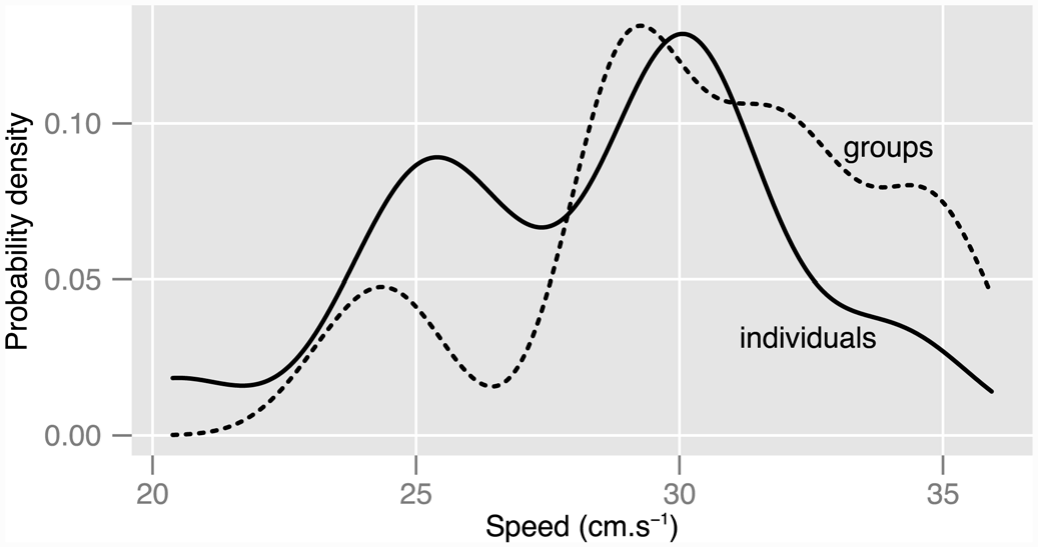
Kernel estimation of the probability density distribution of swimming speeds of larvae recorded when followed by divers. (equivalent to a continuous histogram). A larger proportion of groups swim at fast speeds compared to individuals.

When followed by divers, individuals and groups swim at similar average depths: 5.6±1.6 m and 5.8±1.2 m respectively (mean±SD; t-test, t = −0.5, *p* = 0.6). Within each run, variability in depth is also of similar magnitude: within-run standard deviation of depth is 1.15 m on average for individuals and 1.07 m for groups (t-test, t = 0.7, *p* = 0.4). Groups, however, swim slightly (7%) faster than individuals: 30±3.4 cm s^-1^ for groups vs. 28±3.6 cm s^-1^ for individuals (mean±SD, t-test, t = −2.1, *p* = 0.04). The general shape of the distributions of swimming speeds is similar for individuals and groups (Kolmogorov-Smirnov, D = 0.34, *p* = 0.11), but a larger proportion of groups swim at faster speeds (Fig 3).

### Group orientation

Within each run, groups are more directional than individuals (higher $\bar{r}$ in Table 1). Pooling across locations, the difference in directionality strength between groups and individuals is significant in both diver following (beta regression, *z* = 2.3, *p* = 0.02) and the DISC (*z* = 2.8, *p* = 0.004). Group swimming increases bearing keeping ability by an average of 15%.

**Fig 4:**
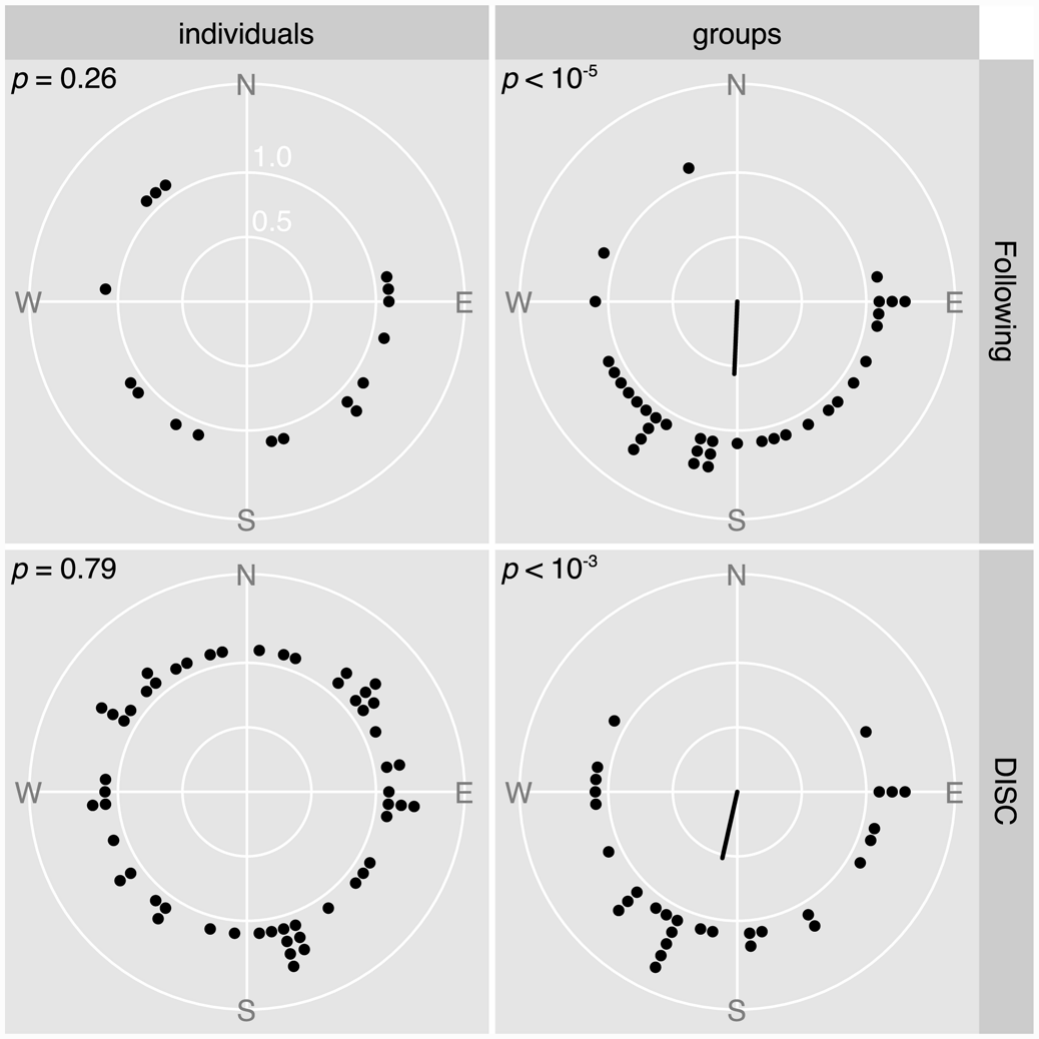
Distribution of within-run mean bearings for individuals or groups (columns) followed or in the DISC (rows). Each dot represents one observation run. When orientation is significant (*p*<0.05, in the corner of panels), the radius in the centre is in the mean direction of orientation and its length represents the precision of orientation (across-runs *r*). Only groups display significant orientation, towards the south.

Groups orient significantly while isolated individuals do not, in all combinations of methods and locations (Table 1). Pooling observations of individuals in the DISC across locations still does not reveal significant orientation despite the increased sample size (Table 1, row 4). Bearings of groups are concentrated in a unimodal pattern around the south, whereas bearings of individuals are more uniformly distributed (Fig 4). The mean southward bearing and overall distribution of bearings of groups are similar between the two observation groups: following on the western side of the island and DISC on the eastern side (Watson-Williams, F = 0.4, *p* = 0.5; Watson, U^2^ = 0.038, *p*>0.1; Fig 4).

*C*. *atripectoralis* larvae are expected to orient cardinally around Lizard Island, independently of location [29], which is consistent with this last result. When tested explicitly, neither individuals nor groups orient towards the Lizard Island coast. Groups on the western side of the island point to the right of the coast (i.e. south) and groups on the eastern side point to the left of the coast (i.e. south also; see S2 Fig for a graphical explanation).

Groups orient also better than do individuals relative to the azimuth of the sun. Furthermore, in most conditions, bearings are more concentrated towards the sun than towards any cardinal direction (S3 Fig). Finally, in the DISC only, directionality is stronger when the sun is low in the sky and its bearing is easier to assess (beta regression of $\bar{r}$ on zenith angle: for individuals, *z* = 3.2, *p* = 0.001; for groups, *z* = 2.89, *p* = 0.004; S4 Fig). Both results suggest the use of the sun as an orientation cue.

## Discussion

These first observations of group orientation behaviour in larval fish show that groups orient more consistently in a common direction, swim on a 15% straighter course (i.e. are better at keeping a bearing), and are 7% faster than isolated individuals. Relative to their environment, groups of *C*. *atripectoralis* orient cardinally and towards the sun rather than in the direction of the nearest coast.

Because orientation seems mediated by solar cues, different solar conditions between observations could have affected the results. However, the median zenith angle is not different between individuals and groups (Wilcoxon, W = 2053, *p* = 0.3; same result when tested within each observation method) and the proportion of non-cloudy skies was high and not significantly different between individuals (91%) and groups (96%; *χ* ^2^ = 0.3, *p* = 0.6). Overall, observations involving various methods, locations, and treatments occurred throughout the day and were spread over non-contiguous days, spanning a short period of steady, calm weather. A systematic bias by environmental conditions such as tides, winds, light, temperature, etc. favouring group runs is therefore extremely unlikely. Furthermore, fish larvae settle in greater numbers at night [41]. Although the observations in this study would be valid for the days preceding settlement, they beg the question: how are fish larvae orienting at night and are they still able to remain in coherent groups? A recent study showed that fish larvae vocalize, which could help them maintain group cohesion even in the dark [42].

Two different observation methods were used: following involves human observers and the larvae are free to move in their three dimensional environment, whereas the DISC relies on automatically captured images of the specimen in a semi-enclosed environment. Both show similar improvements in bearing keeping and overall orientation by groups. Those methods were used in two different environments: the western site is a bay, off the leeward side of the island, with a 18 to 25 m-deep bottom; the eastern site is a more open environment, on the windward side of the island, over a 35 m deep bottom. Yet, the distributions of bearings are similar in both locations, for individuals and groups. The consistency of these results bring a little more generality to the observations of group behaviour; although, of course, observations of other species in other locations are required to assess its global relevance.

A review of similar studies confirmed that orientation of *C*. *atripectoralis* near Lizard island is location-independent and that individuals orient in a southerly direction [29]. Here individuals do not significantly orient in a cardinal direction, only groups do; still, the mean direction is to the south. Previous studies found similar patterns [29] and, in all those cases where no significant orientation was found, the sample size was <25 and may have been too small for the southerly direction to significantly emerge from the noise. In addition, in most other studies, data were collected over a shorter time span within each day than in this one (commonly 4.5 h versus 6.5 h here). If larvae indeed orient using a sun compass, the spread in the sun azimuth was lower in previous studies, which translated into a significant cardinal signal. Here, the spread was larger and the cardinal signal not significant anymore. This is consistent with the result that followed individuals in this study were significantly oriented with respect to the sun’s direction (S3 Fig).

The ecological relevance of these results also depends on how common schooling is, across taxa and throughout ontogeny. Larvae of a number of taxa of fish are observed to swim in groups before settlement, even if the same species do not necessarily school as juveniles or adults (list in [16]). So schooling behaviour could be quite common at settlement-stage. Priori to settlement, larvae of *Gobiosoma bosci* (naked goby) have been observed to swim in groups [43]. In *Pseudocaranx dentex* (striped jack) the onset of schooling seems conditioned by the development of the central nervous system and occurs at about 15 mm in length, after the development of swimming organs (fins rays in particular, which are complete at 9 mm) [44]. In *Aldrichetta forsteri* (yellow-eyed mullet), aggregation occurs at 4–6 mm, when fins are still being formed. Even earlier in development, larvae of some Aulorhynchidae and Trichodontidae are known to school almost immediately after hatching (see [16] for others). No information is available regarding when larvae of *Chromis atripectoralis* begin to swim in groups. They might form groups only when they meet in the light trap, hence limiting the relevance of the observed orientation behaviour. However, each light traps usually contained several hundred fish larvae of various species and many more other organisms (in particular crustacean larvae and annelids), making fine intra-specific interactions unlikely. Only indirect evidence, such as homogeneous otolith microchemistry, suggests cohesion of cohorts in the same water mass (with the same chemical signature) throughout the larval phase, for another Pomacentrid [45] and a Tripterygiid [46]. Outside of fishes, *Sepioteuthis lessoniana* (squid) schools at 30 d after hatching [47]. But overall, information is still mostly lacking [16, 48]. Basic descriptive research is definitely needed regarding ontogeny of schooling in various taxa; this is also true for many other important life history traits during the larval stage of marine species (mortality, swimming abilities, energetics of swimming, etc. [49]).

During the pelagic dispersal phase, larvae swimming in groups would have a shorter swimming path (because they swim in a straighter course), swim faster, and orient more accurately than individual larvae. They would therefore reach their destination more often and more quickly. Faster swimming has an energetic cost and typically results in decreased endurance [50]. On the other hand, leaving the pelagic environment sooner means sustaining less mortality before the critical stage of settlement. Indeed, mortality rates of pelagic fish larvae are very high, in the order of 20% per day [15]. A few days less spent in the water column could therefore affect recruitment rates. Finally, the reduced dispersal time and more accurate orientation would limit the spread of the larval cohort during the pelagic phase. This, in turn, would alter the networks of connection between adult populations of demersal fishes, mostly ensured by larvae. The potential ecological effects of improved orientation in groups on recruitment and connectivity are therefore numerous and should be investigated, for example by implementing the orientation parameters measured here in spatially-explicit dispersal models representing directional swimming through a biased random walk [27].

Two categories of mechanisms can explain improved orientation by groups: either individuals follow a skilled leader or group dynamics correct individual errors and result in a better average choice. These are extremes in a range of possible processes: several leaders can be present [10], individuals with better information than the rest can share it within the group without explicitly leading [4, 11], group dynamics can improve the accuracy of even the best, leading, individuals [8], etc. We did not set out to explicitly test a mechanism here but a number of observations point towards the leaderless end of the spectrum.

First, qualitative observations of groups followed and in the DISC suggest that the individual(s) initiating changes in direction are not always the same. But individuals turn quickly and cross paths often in the group; doing so, they mask each other, which makes it impossible to follow each individual unambiguously for 10 min. Therefore, quantitative data could not be extracted from the images captured by the DISC or from a few videos recorded while following larvae. However, the advent of affordable and user-friendly 3D cameras may help solve these ambiguities by adding another plane of view in which to track each individual.

Second, leader-follower interactions often involve a more knowledgeable leader: a pigeon that already homed along a given path, older migrating birds which know the migration route, etc. In the case of the natal dispersal of marine coastal organisms, no individual has former knowledge of the pelagic environment. Some individuals may be more skilled than others but it is unclear how potential followers could recognise those potential leaders within a group of conspecifics of approximately the same age, size, and stage of development, with no way of checking which orientation direction is the right one until the very end of the larval phase.

Finally, the leaderless case can be explained by the “many-wrongs” hypothesis [3]. In this scenario, all individuals are equally wrong when choosing a direction but bias their choice towards the centre of the group, to ensure group cohesion. When following larvae, we did observe that some individuals would slightly diverge from the main group only to return to it shortly afterwards, which is consistent with this group cohesion mechanism. By doing so, organisms avoid extreme deviations from the true direction; the individual errors cancel out. This mechanisms requires very little cognitive skill: no memory, no recognition of a particular individual in the group, etc. This parsimonious explanation is seductive in the case of young, small, not yet fully developed organisms whose cognitive skills are unknown but probably poorer than those attributed to homing or migrating birds.

Such group dynamics can influence decisions beyond orientation. For example, consensus decision making allows adult *Gasterosteus aculeatus* (stickleback fishes) to better discriminate between various fake environments [51, 52] or *Gambusia holbrooki* (mosquitofish) to react faster and more efficiently to predators [53]. Similarly, very simple social interactions can explain why schools of juvenile fish are much better than individuals at finding shelter in shadows (*Notemigonus crysoleucas*, golden shiner [54]). Finally, even organisms with a less extensive central nervous system, such as *Blattella germanica* (cockroaches), can display effective group foraging behaviour without advanced social interactions [55]. For marine larvae in the relatively featureless open water environment, living in groups could therefore have consequences for survival and foraging success in addition to orientation, even very early during ontogeny because it may not require advanced cognitive skills.

—

To conclude, two observations methods concur that groups of fish larvae orient better than isolated individuals. This extends observations so far restricted to birds. The generality and mechanism of these improved orientation abilities are not yet known for fishes. But the mechanism could require very little cognitive ability and generalise to enhance survival and foraging, in addition to habitat finding. Such improved abilities would have important consequences for dispersal trajectories and habitat selection, during a stage of key ecological importance for coastal marine populations.

## Supporting Information

S1 Fig Engineering rendition of the Drifting In Situ Chamber (DISC). To save space, the full length of the line and the bottom half of the drogue are not represented.
(PDF)

S1 Video Excerpt from a video shot while following a group of about 17 larvae during a test run (1 min 30 s), in open waters off Lizard Island. Larvae start as a small ball-shape shoal and quickly spread along the horizontal to take the typical shape described in Fig 2. At the 1:10 mark, one larva begins to lag behind the rest of the group before finally moving out of the frame. Video by C. Paris and R. Paris, November 2013. https://dl.dropboxusercontent.com/u/1047321/group-following-SI2.mp4.
(MP4)

S2 Fig Distribution of within-run mean bearings relative to the direction of the coast (left) and distributions for groups shown in their geographical context (right). Each dot represents one observation run. When orientation is significant (*p*<0.05, in the corner of panels), the radius in the centre is in the mean direction of orientation and its length represents the precision of orientation (across-runs *r*). Individuals do not display significant orientation. Groups on the west side of the island swim to the right of the coast (i.e. south). Groups on the east side of the coast swim to the left of the coast (i.e. south again). This suggests a cardinal, southward, orientation rather than an orientation relative to the coast.
(PDF)

S3 Fig Distribution of within-run mean bearings relative to the direction of the sun (i.e. the sun’s azimuth). Each dot represents one observation run. The concentration of mean bearings (across-runs *r*) and its significance (Rayleigh’s *p*) are indicated in the corner of each panel. When orientation is significant (*p*<0.05), the radius in the centre is in the mean direction of orientation and its length represents the concentration of mean bearings, i.e. the precision of orientation (across-runs *r*). Individuals in the DISC do not display significant orientation. Followed individuals and groups orient towards the sun. Bearings are more concentrated relative to the sun than relative to a cardinal direction (compare values of *r* with Table 1) and the mean direction is more consistent among treatments and techniques (compare the direction of the radii with Fig 4). This suggests that larvae use the sun as an orientation cue.
(PDF)

S4 Fig Strength of directionality (within-run *r*) as a function of sun elevation (zenith angle). Each point is an observation run. Lines are beta regression predictions (solid when significant, dashed when not). Shaded areas represent the inter-quartile range for the regression line. In the DISC, where *r* values are more variable [29], *r* significantly increases when the sun is lower in the sky (larger zenith angle) and its direction is easier to detect.
(PDF)

S1 Data Data of the study. as a Comma Separated Values (.csv) file. Columns are described in the first 20 lines. Column names are at line 21. Data starts at line 22.
(CSV)
