# Supplementary figures and images for: With a Little Help from My Friends: Group Orientation by Larvae of a Coral Reef Fish
Source: PLoS One. 2015 Dec 1;10(12):e0144060. doi: 10.1371/journal.pone.0144060 (PMC4666641; doi:10.1371/journal.pone.0144060)

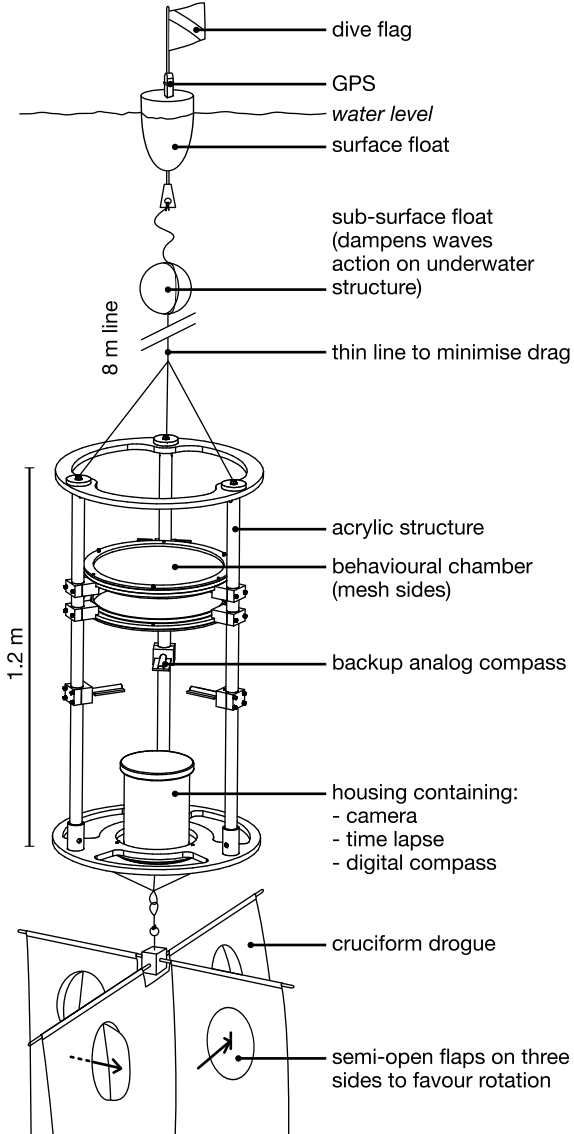

Supplement: S1 Fig — To save space, the full length of the line and the bottom half of the drogue are not represented. (PDF) [file pone.0144060.s001.pdf]

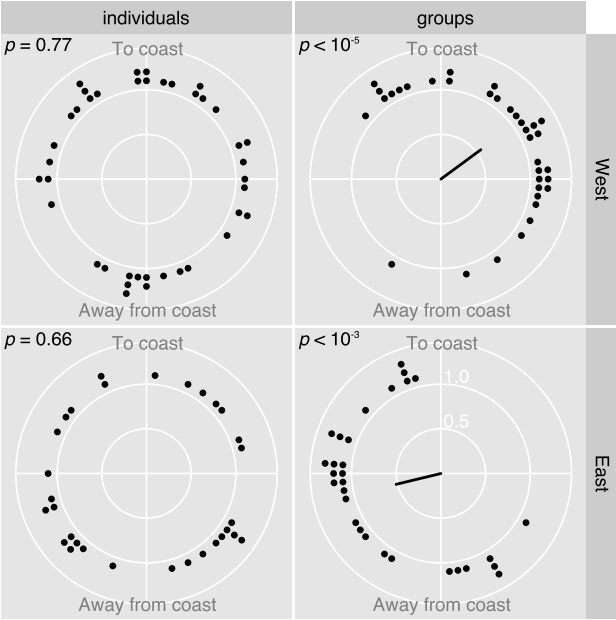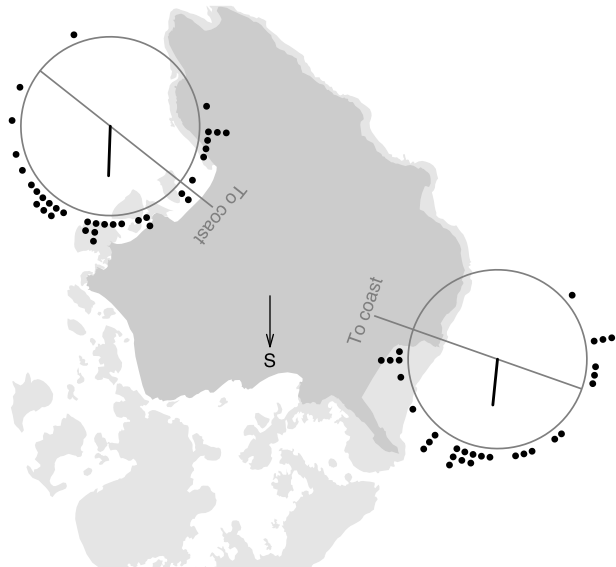

Supplement: S2 Fig — Each dot represents one observation run. When orientation is significant (p<0.05, in the corner of panels), the radius in the centre is in the mean direction of orientation and its length represents the precision of orientation (across-runs r). Individuals do not display significant orientation. Groups on the west side of the island swim to the right of the coast (i.e. south). Groups on the east side of the coast swim to the left of the coast (i.e. south again). This suggests a cardinal, southward, orientation rather than an orientation relative to the coast. (PDF) [file pone.0144060.s003.pdf]

individuals

$r = 0.42$   
 $p = 0.046$

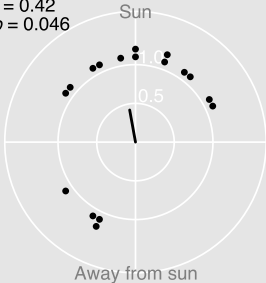

groups

$r = 0.37$   
 $p = 0.007$

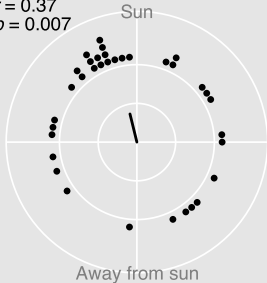

Following

$r = 0.15$   
 $p = 0.29$

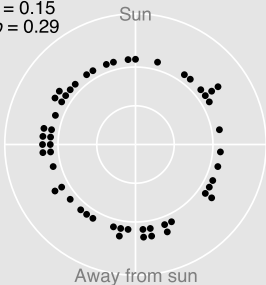

$r = 0.6$   
 $p < 10^{-5}$

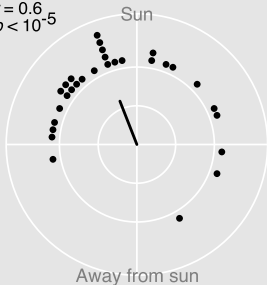

DISC

Supplement: S3 Fig — Each dot represents one observation run. The concentration of mean bearings (across-runs r) and its significance (Rayleigh’s p) are indicated in the corner of each panel. When orientation is significant (p<0.05), the radius in the centre is in the mean direction of orientation and its length represents the concentration of mean bearings, i.e. the precision of orientation (across-runs r). Individuals in the DISC do not display significant orientation. Followed individuals and groups orient towards the sun. Bearings are more concentrated relative to the sun than relative to a cardinal direction (compare values of r with Table 1) and the mean direction is more consistent among treatments and techniques (compare the direction of the radii with Fig 4). This suggests that larvae use the sun as an orientation cue. (PDF) [file pone.0144060.s004.pdf]

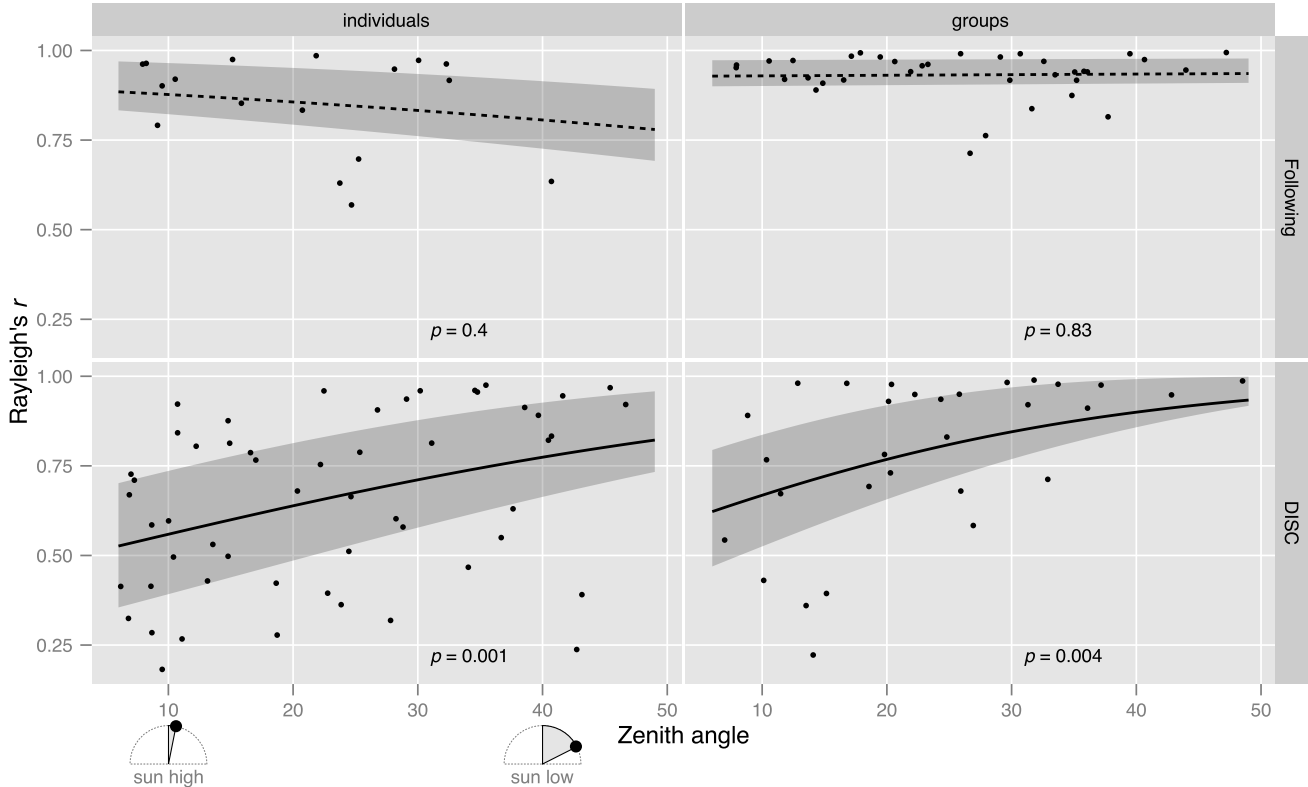

Supplement: S4 Fig — Each point is an observation run. Lines are beta regression predictions (solid when significant, dashed when not). Shaded areas represent the inter-quartile range for the regression line. In the DISC, where r values are more variable [29], r significantly increases when the sun is lower in the sky (larger zenith angle) and its direction is easier to detect. (PDF) [file pone.0144060.s005.pdf]
